# Irregular Tremulous Movements and Infrequent Seizures: A Clinical-Electrophysiological Diagnosis of Benign Adult Familial Myoclonus Epilepsy

**DOI:** 10.7759/cureus.56303

**Published:** 2024-03-17

**Authors:** Kazuki Imon, Shuichiro Neshige, Akiko Maeda, Yumiko Yamamoto, Hirofumi Maruyama

**Affiliations:** 1 Department of Clinical Neuroscience and Therapeutics, Hiroshima University, Hiroshima, JPN

**Keywords:** giant somatosensory evoked potential, myoclonus, perampanel, essential tremor, benign adult familial myoclonus epilepsy

## Abstract

We report a case involving a 31-year-old male without any known precipitating injuries presenting with involuntary finger movements and rare seizures. There was a noted family history of tremulous movements. Yet the characteristics of his finger movements were irregular and not typical of essential tremor (ET). Electrophysiological examinations, including video EEG, showed no epileptic discharges, and brain MRI results were normal. However, somatosensory evoked potentials (SEP) revealed the presence of giant SEP, and a positive cortical (C)-reflex was observed, leading to a clinical diagnosis of benign adult familial myoclonus epilepsy (BAFME). Management with valproic acid and perampanel resulted in a significant reduction of symptoms. This case highlights the necessity of considering BAFME in the differential diagnosis for atypical tremorous finger movements, especially with a relevant family history, and the critical role of electrophysiological findings indicative of cortical hyperexcitability.

## Introduction

In the spectrum of involuntary movements, tremors are commonly observed. Central sources of tremor include the cerebellum, red nucleus, olive nucleus, thalamus, and basal ganglia. Thus, tremors can be visible in various conditions. For example, lesions of the cerebellum can also produce a slow tremor during action or intention, not contemplation. Conversely, tremors in Parkinson's disease thought to be due to dysregulation of the basal ganglia, present as moderately fast tremors at rest. Besides, essential tremor (ET) represents one of the most common disorders exhibiting a tremor with an estimated prevalence ranging from 2.5%-10% within the general population [1]. The age distribution of the onset of ET shows a bimodal pattern, in the twenties and sixties. Additionally, a characteristic feature involves the presence of a family history [2]. Specifically, the proportion of ETs with a family history is reported to be between 17.4% and 100% [3]. Consequently, young and young adult patients presenting with tremors who have a family history are often provisionally diagnosed with ET in clinical settings. However, in such a setting, the differential diagnosis includes benign adult familial myoclonus epilepsy (BAFME) [4] [5], which encompasses cortical tremors and may present similar challenges in clinical distinction from ET. The complexity of diagnosis is further compounded by the shared familial trait between ET and BAFME, necessitating a thorough clinical and electrophysiological evaluation to reach a definitive diagnosis. Herein, we report a case that was initially diagnosed with ET but was later reclassified as BAFME based on the unique presentation of tremors and specific electrophysiological findings.

## Case presentation

This is the case of a 31-year-old male who had no history of central nervous system infection, febrile convulsions, head trauma, or perinatal abnormalities. His familial history was notable for his mother experiencing pre-sleep tremulous movements of the hand commencing at approximately 40 years of age; however, the presence of epileptic seizures in her medical history remains undocumented.

At the age of 25, he began to experience tremulous movements in his limbs and fingers at bedtime, which insidiously progressed to intermittent daytime occurrences. Thus, he visited a local hospital and was diagnosed with ET, warranting observational management. At the age of 27, the frequency of his tremulous movements increased, and at 31, he had an initial unprovoked convulsive seizure characterized by spontaneous left conjugate eye deviation, ipsilateral head version, and tonic-clonic seizures involving bilateral limbs, resolving spontaneously after about two minutes. Thus, he was transported to the local hospital. After administering intravenous anti-seizure medication, he was started on valproic acid therapy. Although the intensity of tremulous movements was mildly decreased, he was introduced to our institution for a comprehensive evaluation of involuntary movements and seizures.

**Figure 1:**
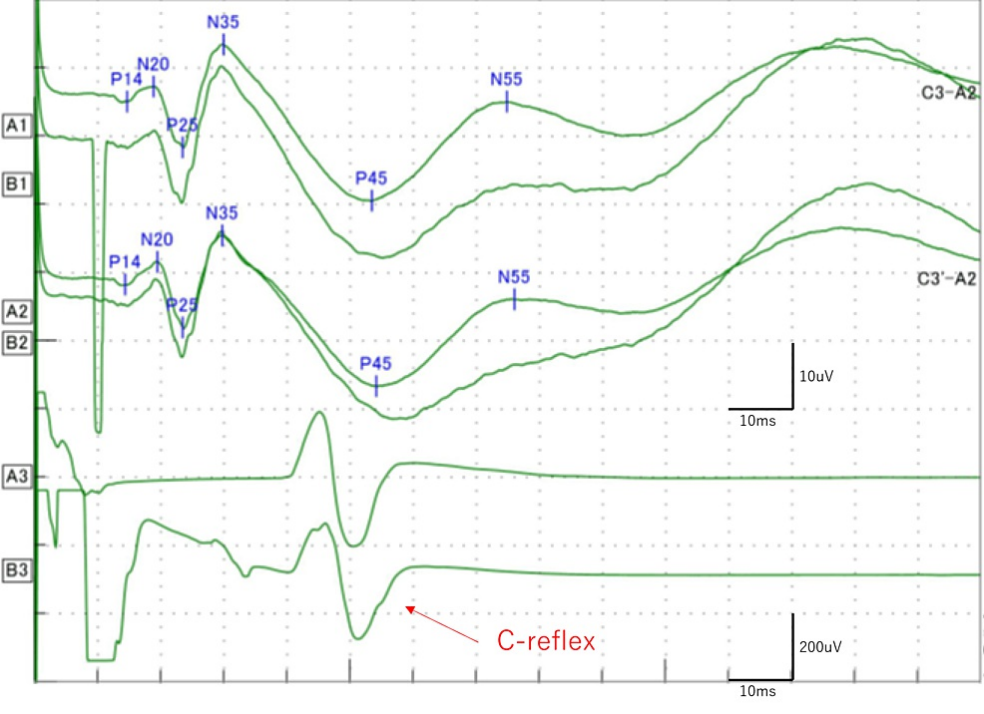
Long-latency somatosensory evoked potentials (SEP) Long-latency SEP reveals augmented responses, with an N20-P25 amplitude of 8.91μV and a P25-N35 of 15.08μV, i.e., giant SEP. The cortical (C)-reflex is also visible following the ipsilateral median stimulation.

On examination, the level of consciousness was normal, and no neurological abnormalities were visible, although irregular, fine dysrhythmic finger movements were visible in postural maintenance even under the administration of valproic acid (600 mg/day). Blood tests revealed unremarkable findings and brain CT and MRI showed no structural abnormalities. Tests for cognitive impairment (Wechsler Adult Intelligence Scale-IV; intelligence quotient range: 94-114) showed normal cognition. Video EEG monitoring revealed no epileptic discharges but occasional generalized theta waves during awakeness. However, the long-latency somatosensory evoked potentials (SEP) test revealed augmented responses, with an N20-P25 amplitude of 8.91μV and a P25-N35 of 15.08μV, thus satisfying the criteria for giant SEP (Figure 1) [6].

The cortical (C)-reflex was also noted during the SEP examination. Based on these clinical-electrophysiological findings, he was diagnosed with BAFME. Therapeutic management with valproic acid resulted in significant weight gain, necessitating a dose reduction from 600 mg to 200 mg. Besides, an adjunctive treatment with perampanel at a dosage of 2 mg/day was introduced. This regimen led to a substantial remission of tremulous movements, with no subsequent epileptic seizures reported.

## Discussion

We herein report the clinical narrative of a male patient in his late twenties, originally diagnosed with ET. However, his clinical progression and electrophysiological assessments ultimately delineated a diagnosis of BAFME. Distinguished from the rhythmic oscillations typical of ET, this patient's postural tremulous movements were irregular, dysrhythmic, and subtle in nature. A therapeutic regimen combining valproic acid with low-dose perampanel demonstrated efficacy in controlling cortical myoclonus and epileptic convulsions. 

Benign adult familial myoclonus epilepsy is an autosomal dominant epilepsy syndrome presenting with cortical myoclonus and infrequent generalized tonic-clonic seizures in adulthood [7, 8]. The disorder is relatively uncommon, with a reported prevalence of one in 3,500 individuals [9]. Clinical manifestations are predominantly characterized by tremor-like myoclonus and generalized tonic-clonic seizures. While initial symptomatology is typically mild and progresses languidly, there are instances of symptom exacerbation with advancing age [10]. The diagnosis is predicated upon a suite of criteria: 1) autosomal dominant inheritance; 2) cortical tremor; 3) infrequent generalized and focal seizures; 4) features of cortical reflex myoclonus demonstrated by electrophysiological studies; 5) lack of evident cognitive decline, cerebellar ataxia, and other neurological symptoms at least in the early stage of the clinical course; 6) lack of clear progression of cortical tremor, which impairs daily activity in the early stage of the clinical course [11]. Thus, although a genetic test was not evaluated, our case was clinically diagnosed with BAFME. Additionally, there was a potential family history in our case. The onset of the disease was probably in the proband's twenties and the forties for the mother. Thus, there might be anticipation, which is consistent with the genetic characteristics of BAFME [12].

The genetic etiology of BAFME has been traced to the pathological elongation of TTTCA/TTTTA repeats within the intron regions of genes including SAMD12, TNRC6A, and RAPGEF2 [13]. Although genetic testing facilitates a definitive diagnosis, it may not be readily accessible to all patients, thus elevating the importance of electrophysiological evaluation. Therefore, vigilant application of electrophysiological testing to identify giant SEP is instrumental in diagnosing BAFME. Additionally, screening based on differences in tremors is necessary to avoid misdiagnosis as an ET. Thus, meticulous assessment of tremulous movements is vital to differentiate BAFME from ET, as irregular dysrhythmic patterns were evident in the present video documentation. Additionally, the potential progression of cortical activity associated with BAFME as one ages [14] is also significant in distinguishing it from ET. To rule out other differentials of myoclonus epilepsy, conducting whole-exome sequencing was necessary. However, we did not conduct it as the present case had a normal cognition level and the clinical presentation was stable after the initiation of anti-seizure medication. 

The standard management for BAFME involves the administration of antiepileptic drugs. In the present case, valproic acid was the initial medication but was reduced due to adverse effects, and perampanel was added. Perampanel provided a successful therapeutic outcome in the present case, likely attributed to its ability to suppress cortical hyperexcitability [15]. 

## Conclusions

We experienced a case of BAFME diagnosed through medical history, examination findings, and electrophysiological tests. Reconsidering the initial diagnosis of ET led to the administration of appropriate medication, resulting in improved symptoms. Thus, when tremulous movements are present alongside a family history, BAFME should also be considered in the differential diagnosis. This necessitates the confirmation of specific electrophysiological findings indicative of cortical excitability. Consequently, initiating treatment with drugs that can suppress cortical excitability is crucial.
